# Predictors of shunt response in iNPH: differentiating responders, early and late non-responders, and the role of valve adjustments

**DOI:** 10.1007/s00701-025-06663-9

**Published:** 2025-09-13

**Authors:** Santhosh G. Thavarajasingam, Mahmoud El-Khatib, Stefania Roxana Kalb, Ahmed Salih, Daniele S. C. Ramsay, Ahkash Thavarajasingam, Dragan Jankovic, Malte Ottenhausen, Darius Kalasauskas, Andreas Kramer, Angelika Gutenberg, Florian Ringel

**Affiliations:** 1 Department of Neurosurgery, LMU University Hospital, LMU Munich, Munich, Germany; 2 Imperial Brain and Spine Initiative, Imperial College London, London, UK; 3 Department of Neurosurgery, University Medical Center Mainz, Mainz, Germany

**Keywords:** NPH, Shunt-response, Non-responders, Shunt-adjustment

## Abstract

**Introduction:**

Idiopathic normal pressure hydrocephalus (iNPH) is diagnosed based on a positive shunt response. However, up to 40% of patients who undergo ventriculoperitoneal (VP) shunting fail to exhibit sustained improvement. The management of iNPH remains challenging, particularly for non-responders who deteriorate despite surgery. We aimed to determine what features differentiate between long term versus short term responders and do valve adjustments affect their outcome?

**Material and methods:**

We included patients that underwent ventriculoperitoneal shunt surgery for iNPH between December 2006 and December 2016. Patients were stratified as early (< 6 months) and late (> 6 months) non-responders, and responders. Descriptive statistics, time series plotting, chi-squared tests, and ANOVA analyses were used.

**Results:**

Our cohort of 65 iNPH patients exhibited a mean follow-up of 3.75 years and consisted of 53.8% early non-responders, 15.4% late non-responders, and 30.8% responders. Comorbidities were distributed across all groups but did not significantly differentiate between response categories. A considerable subset experienced symptom deterioration after the six months mark. Shunt valve adjustments were more frequent in non-responders but did not prevent continued deterioration. In late non-responders, valve adjustments merely slowed symptom progression, without halting deterioration.

**Conclusion:**

Our study underscores that valve pressure adjustments in early non-responders, who likely never benefit from shunt surgery, are not effective, and highlights the emergence of a late non-responder phenotype, where symptom deterioration becomes evident 6 months post-shunting. Our findings outline the need to explore alternative treatment strategies for managing symptoms in iNPH non-responders, as well as prolonged follow-up regimens to monitor late non-responders.

## Introduction

Early diagnosis and optimized treatment for idiopathic normal pressure hydrocephalus (iNPH), has become increasingly important with its disproportionately increased prevalence in the elderly population [1, 13]. The prevailing gold standard to achieve this continues to be ventriculoperitoneal (VP) shunting, a surgical intervention involving the diversion of cerebrospinal fluid [11]. However, the heterogenous response to VP shunting means predicting shunt response and selecting surgical candidates correctly remains a clinical challenge.

Recent meta-analyses by Thavarajasingam et al. [26–28] have identified clinical, biochemical and radiological predictors of shunt response [26, 28]. These findings highlight the potential utility of intraparenchymal intracranial pressure (ICP) monitoring, callosal angle assessment, and periventricular changes as predictive factors for shunt response, alongside neurological assessment including detailed cognitive assessment and gait analyses. However, primary evidence beyond the usual 12-months follow-up mark on long term shunt responsiveness remains sparse.

Some literature, albeit minimal, exploring long term follow-up has suggested secondary deterioration many years after shunting, in patients who were initially shunt responsive, giving rise to the phenomenon of delayed shunt non-responders (S-NR). In a retrospective study conducted by Pujari et al., it was noted that a substantial proportion of patients experienced secondary deterioration approximately 2.7 years post VP shunt insertion, leading to the need for pressure level management or shunt revisions [24]. Additionally, a majority of patients monitored for at least three years in a comparable study by Gutowski et al. needed shunt revisions [10].

Predictors of late shunt non-responders (S-NR) remain insufficiently explored. Given the patient burden and the risks associated poorly managed iNPH, a comprehensive insight into the prognostic indicators of delayed S-NR is needed [19, 23]. This study focuses on the emergence of a distinct late non-responder phenotype, in which symptom deterioration occurs primarily after the six-month mark.

## Methods

This retrospective single centre cohort study was conducted using electronic patient records from December 2006 to December 2016. It aimed to evaluate the shunt response of all patients of a large tertiary care neurosurgical center, that underwent ventriculoperitoneal shunting (VP), with extended follow-up.

### Selection of patients

Internationally recognised guidelines were used to select patients requiring VP shunting [22]. Any patient with one of Hakim’s triad underwent cranial imaging (CT or MRI), patients with recognised features of NPH such as Evan’s Index > 0.3, calossal angle between 40–90 degrees etc.^11^ were selected to undergo a CSF Tap test; on average, 38mL ± 13.4mL of CSF was drained. Subsequently all patients underwent a battery of assessments both pre and post tap test including Romberg standing test, the Unterberger stepping test, the rope walking test and the Timed Up and Go (TUG) test, orientation to time, place and person, the Mini-Mental Status Test and the DemTect Test. Patients who improved in the mobility tests post CSF tap underwent VP shunting. Improvement was assessed one hour after tap test and defined as subjective improvement in gait tests or faster TUG time and improvement of greater or equal to one point in MMSE. Drainage systems from Christoph Miethke GmbH & Co and Integra LifeSciences, the manufacturers of the Codman Hakim valve, were used. Patients then underwent cranial imaging (CT vs MRI) post operatively.

### Data collection

Data collection, completed by 2022, involved storing and analyzing anonymized patient information. Ethical approval was obtained from the hospital’s ethics board and head of department, inline with local protocol.

Patients were followed up at 6–12 month intervals after surgery following their initial 3 month follow-up. Patients with no follow-up after 3 months were excluded from this study. At each follow-up period the following was recorded: days between surgery and follow-up, complications since last follow-up including; hospitalisation, alternative NPH diagnosis made, cerebral infarction, fall with cranial bleed. Additionally, assessment of improvements of Hakims triad symptoms were rated as + 1 (improved), 0 (same), −1(worsened) and were recorded, this was in relation to their previous appointment, not surgery.

Any valve pressure changes and subsequent improvement of symptoms and cranial imaging findings were noted. Shunt patency was tested by checking if the shunt pump was still “pumpable”. We then checked for shunt dislocation or disconnection by using CT head with 3D reconstruction as well as AP and lateral X-rays of the skull neck thorax and abdomen. Complications revealed by cranial imaging, e.g. signs of over-drainage, under-drainage hygroma, haemorrhage and any intervention required were also recorded.

### Statistical analysis

Calculations were performed using R (version 4.0.4), with a significance threshold set at p < 0.05. Exploratory data analysis and time-series analyses was conducted to examine the data's underlying structure and patterns. We used a mathematical t_1/2_ framework to categorize iNPH patients post-surgery into distinct responder types, informed by temporal symptom change patterns. Our methodology entailed the calculation of the final cumulative symptom change. Each scoring of the patients of their clinical status as described above, was scored as + 1 for “improved”, 0 for “same” and −1 for “worse”. The cumulative symptom status was the sum of these scores after all follow-ups of the respective patients. This was followed by the identification of the time point at which each patient experienced a 50% decline in symptoms. Utilizing these metrics, we assigned patients to one of three categories: "Responder", "Early Non-Responder (< 6 months)", or "Late Non-Responder (> 6 months)". The proportional representation of non-responders at each time point was then calculated to provide a comprehensive understanding of the treatment outcomes over time. Chi-square and ANOVA tests were used to evaluate differences in the chosen covariates between three response groups, namely: responders, early non-responders (< 6 months) and late non-responders (> 6 months).

## Results

A cohort of 65 patients who underwent VP shunting was observed, with a mean follow-up duration of 3.75 years. Eight patients were excluded as they did not attend 3 month follow-up for unknown reasons, however they did not sustain complications.

### Temporal analysis

**Fig. 1:**
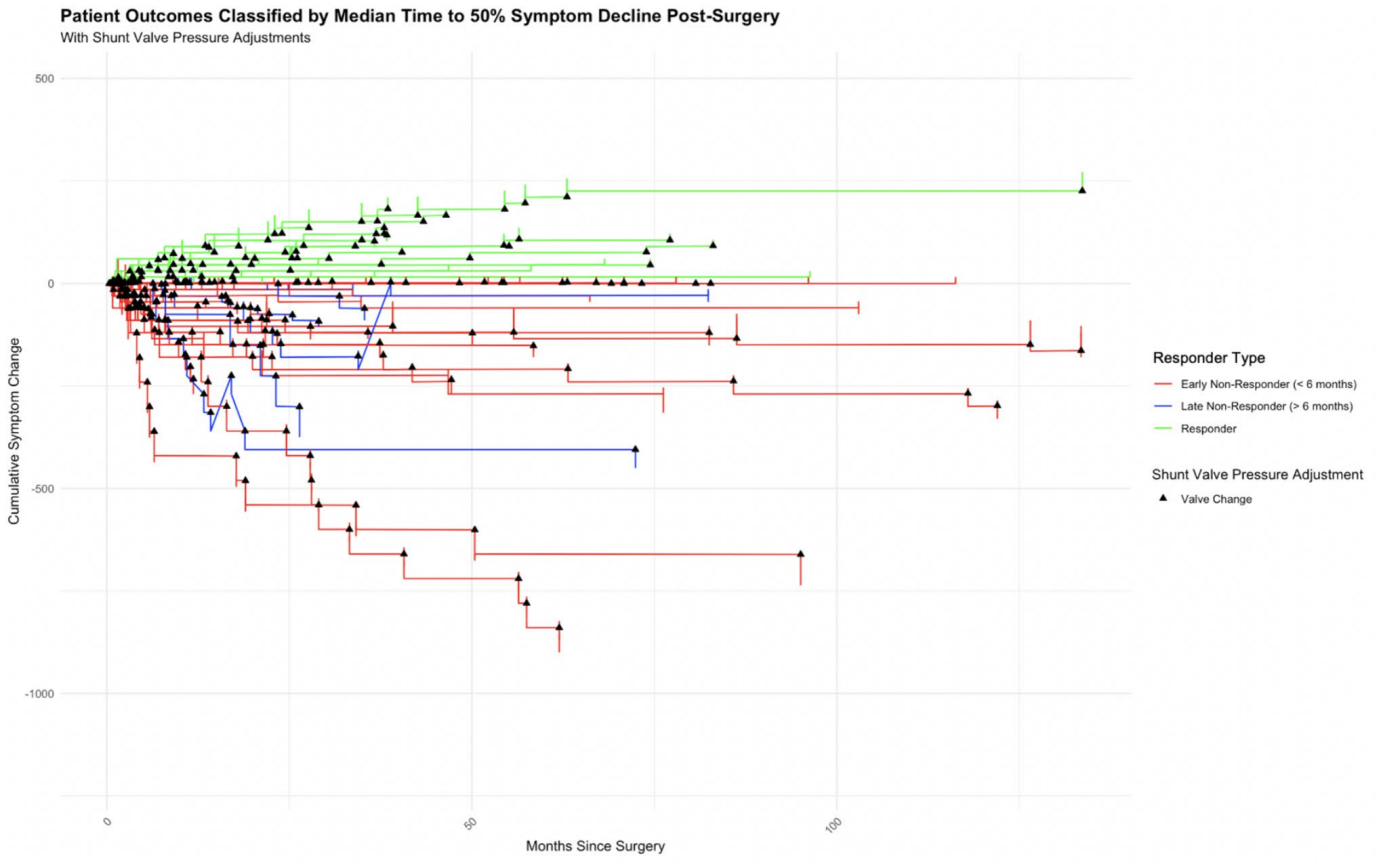
Longitudinal patient-level symptom change post-ventriculoperitoneal shunt surgery. The lines are grouped into response groups: early non-responder (< 6 months), late non-responder (> 6 months), responder. A black triangle indicates that at the respective time point, the shunt pressure valve was adjusted at the follow-up appointment

Figure 1 illustrates the range of patient symptom trajectories following ventriculoperitoneal shunt surgery for iNPH. The graph tracks cumulative symptom changes over time, with individual patient paths delineated. The analysis identifies a pattern where a subset of patients, categorized as early non-responders, represented by the red lines, exhibit a lack of symptom improvement shortly after surgery. Conversely, a distinct group emerges, labeled as late non-responders (blue lines), where symptom decline becomes more pronounced after the initial six-month period, pointing to a delayed onset of symptom deterioration. The responders, indicated by green lines, generally maintain or improve their symptomatology over the observed timeline.

Following this analysis patients were categorized into three response groups; early non-responders (within the first 6 months) who comprised 53.8% of the cohort. Late non-responders (occurring between 6 months to 3 years), constituted 15.4% of the patient group. Responders to the treatment represented 30.8% of the total sample. Detailed patient demographics, clinical characteristics, and disease-specific attributes across these response categories are compiled in Table 1.

**Table 1:** Summary of clinical and disease characteristics across response categories in our patient cohort. Figure 1 depicts the cumulative symptom change over time for each patient following ventriculoperitoneal shunt surgery. Each line represents an individual patient's trajectory, with the x-axis indicating the number of days since surgery and the y-axis representing the cumulative change in symptoms. The symptom change is calculated by summing the directional changes in symptoms, with improvements, no change, and deteriorations weighted as + 1, 0, and −1, respectively

Group	Early Non-responder (< 6 month)	Late Non-responder (> 6 months)	Responder
Count (% of overall sample)	35 (53.8%)	10 (15.4%)	20 (30.8%)
**Comorbidities**			
CVS risk	97	100	95
Polyneuropathy	9	20	25
Dementia	94	100	85
Other cause of incontinence	9	0	5
Epilepsy	3	10	0
Metabolic disease	43	50	55
Dementia	14	20	5
Malignancy	14	20	10
Psychiatric illness	20	0	10
Prostate disease	17	30	15
Stroke	17	20	10
**Gait impairment**			
Broad-based gait	26	30	30
Small steps	74	90	75
Tendency to fall	11	10	5
Unsteady walking	54	30	45
Directional unsteadiness	11	0	5
Increased turning steps	17	30	10
Mobility aid	0	10	15
Romberg test positive	17	30	10
**Urinary incontinence & miscellaneous**			
Headache	9	20	5
Urinary Incontinence	89	100	80
Speech impairment	17	10	5
Psychomotor slowing	23	10	20
Behavioural issues	3	0	5
Cranial nerve deficit	11	0	0
**Cognition**			
Time disorientated	29	20	0
Globally disorientated	3	0	10
Reduced MMSE	6	0	0
Short term memory impaired	9	10	5
Situationally disorientated	17	10	0
Spatially disorientated	0	20	5
Abnormal Demtect	29	20	0
**Radiological findings**			
Expanded external CSF spaces	17	20	10
Expanded internal CSF spaces	66	80	85
Global brain atropy	14	0	10
**Shunt**			
Abdominal catheter downright	74	70	85
Hakim Medos	37	70	35
ProGav Miethke	63	30	65
Shunt Assistant	54	20	70
**Post-LP improvement**			
Walking improved slightly post-LP	6	20	10
Walking improved clearly post-LP	6	0	10
Improvement in MMSE Post-LP	3	0	10
Improvement in DemTect Post-LP	3	10	0
**Continuous variables**			
Age at surgery (SD)	73.6 (6.8)	77.2 (4.8)	71.3 (7.2)
Symptom duration before presentation in days (SD)	16.2 (15.9)	13.5 (11.8)	20.1 (22.9)
Duration between initial presentation and surgery in days (SD)	96.6 (326.1)	38.2 (59.2)	47.4 (82.3)
Shunt pressure level in mmH2O (SD)	93.4 (23.4)	107 (22.1)	99.5 (23.1)

Table 1 presents a detailed breakdown of clinical characteristics and their prevalence across five defined response categories in patients with idiopathic normal pressure hydrocephalus (iNPH) after ventriculoperitoneal shunt surgery. The groups include early non-responders (within 6 months, late non responders > 6 months responders, and responders. The count and percentage of the overall sample for response category are provided, the rest of the covariates are depicted as percentages offering a snapshot of the distribution within the cohort. Continuous covariates are presented as mean values with SD in brackets () at the bottom

### Patient characteristics & comorbidities

**Fig. 2:**
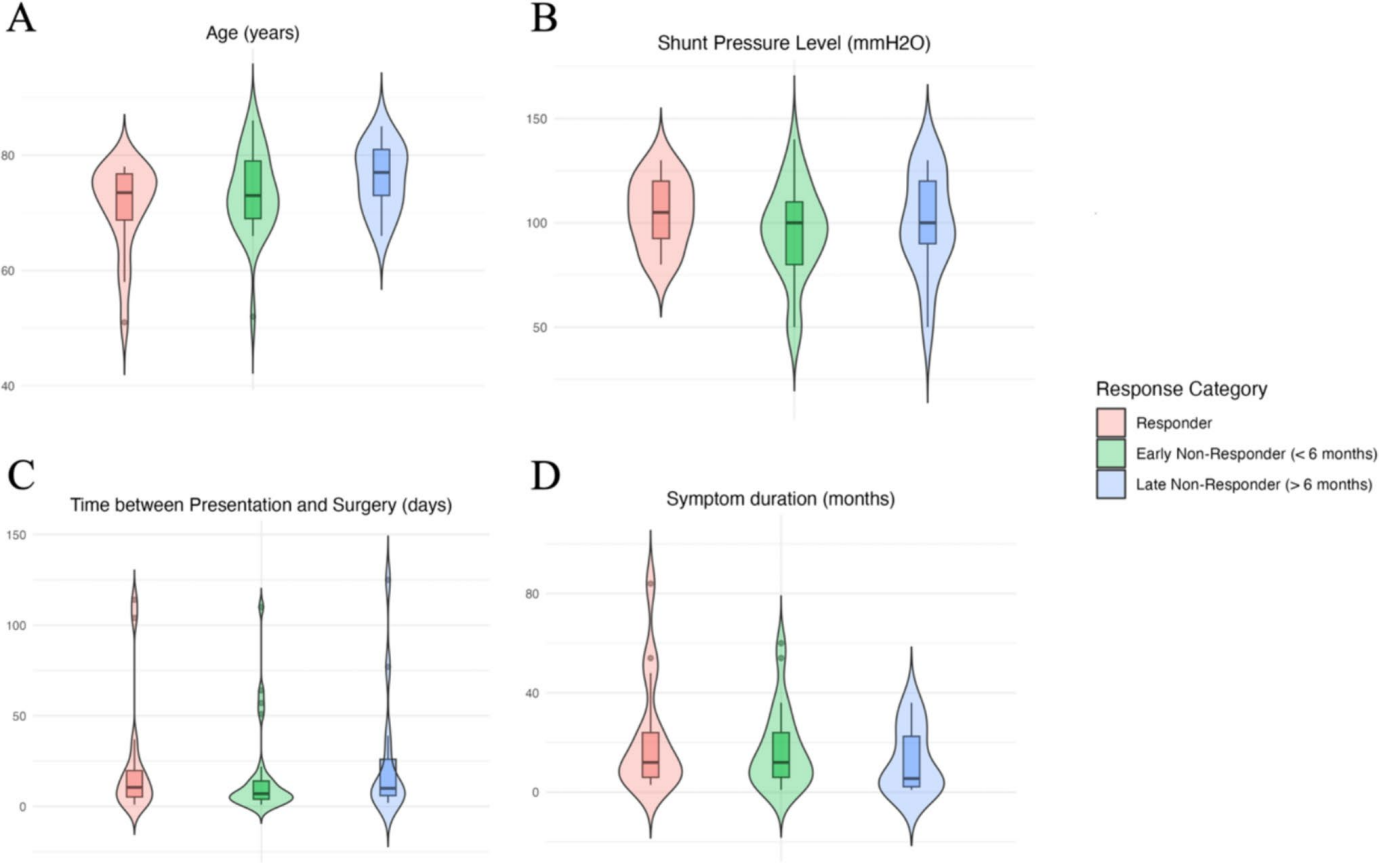
Comparative analysis of continuous covariates by response category post-ventriculoperitoneal shunt surgery. Figure 2 depicts a panel consisting of four sub-figures 2 **A**-**D**, each respectively labelled alphabetically. Figure 2A presents patient age at the time of surgery in years. The distribution of ages is shown by the breadth of the violins, with wider areas indicating a higher density of patients. Figure 2B depicts the shunt pressure level measured in mmH2O. The varied widths of the violin plots reflect the frequency of pressure levels observed among the patients. Figure 2C displays the time interval between initial presentation and surgery in days. The shape of each violin plot suggests the variability in waiting times before surgery. Figure 2D illustrates the distribution of symptom duration prior to surgery across different response categories. Violin plots represent the spread and density of symptom duration in months. Each colour corresponds to a specific response group (early non-responder (< 6 months), late non-responder (> 6 months), responder). The boxplot within each violin shows the median and interquartile range, providing a summary statistic of central tendency and dispersion

An analysis of age at surgery reveals that early non-responders (mean age: 73.6 years, SD: 6.8) were, on average, younger than late non-responders (mean age: 77.2 years, SD: 4.8). The mean age of responders was 71.3 years (SD: 7.2). A visual representation is shown in Fig. 2A where a discernible trend suggests that early responders tend to be younger compared to their non-responding counterparts. The age distribution among late non-responders exhibits a skew towards older individuals, implying a potential age-related gradient in response to surgery. However, ANOVA analysis of age seen in Table 2 does not show any significance between the three groups.

**Table 2:** ANOVA results assessing statistical significance for difference of continuous variables between shunt responders, early non-responders and late non-responders

	*p*-value
Age at surgery	0.13
Shunt pressure level (mmH2O)	0.49
Time between presentation and surgery	0.60
Symptom Duration	0.65

Table 2 delineates the ANOVA results for continuous variables analyzed against the response categories of iNPH patients post-ventriculoperitoneal shunt surgery. These categories range from early non-responders (within 6 months) to late non-responders (> 6 moths) and responders

Comorbidities were broadly distributed across the response categories, with cardiovascular risk factors present in 97% of early non-responders, 100% of late non-responders, and 95% of responders. Dementia prevalence was notably high in all late non-responders (100%) and less so in responders (85%). Neither cardiovascular risk factors, psychiatric history or dementia showed a significant association with the surgical response in this analysis. 3B visually displays metabolic diseases are more frequently observed in late non-responders compared to early non-responders.

The Chi-Squared test results depicted in Table 3 demonstrates the associations between various categorical variables and the response categories of iNPH patients post ventriculoperitoneal shunt surgery, of which only time disorientation was significantly different between the three groups.

**Table 3:** Chi-Squared test results assessing statistical significance for difference of categorical variables between shunt responders, early non-responders and late non-responders

Chi-Squared Test Results for Categorical Variables	
Variable	*p*-value
**Symptoms and co-morbidities**	
Presence of Urinary incontinence	0.34
Presence of Dementia	0.25
Presence of Headache	0.65
Presence of Cardiovascular risk factors	0.71
Presence of Stroke	0.66
Presence of Previous dementia	0.35
Presence of Epilepsy	0.50
Presence of Previous psychiatric illness	0.82
Presence of Prostate disease	0.54
Presence of Diseases causing incontinence	0.96
Presence of Metabolic diseases	0.38
Presence of Comorbidities polyneuropathy	0.33
Presence of Previous malignant disease	0.62
**Gait analysis**	
Walking test: Presence of Small steps	0.26
Walking test: Presence of Mobility aid	0.36
Walking test: Partial Improvement post LP	0.15
Walking test: Substantive improvement post LP	0.49
Walking test: Presence of Unsteady gait	0.18
Walking test: Presence of Tendency to fall	0.61
Walking test: Presence of Broad based gait	0.94
Walking test: Presence of Increased turning steps	0.28
Walking test: Romberg positive	0.09
Walking test: Presence of Directional unsteadiness	0.71
**Neuropsychological analysis**	
Neuropsychological testing: Person disorientated	0.37
Neuropsychological testing: Spatially disorientated	0.17
** Neuropsychological testing: Time disorientated**	0.04*
Neuropsychological testing: Situation disorientated	0.83
Neuropsychological testing: Short term memory issue	0.32
Neuropsychological testing: Presence of Behavioural issue	0.71
Neuropsychological testing: Presence of Speech issues	0.25
Neuropsychological testing: Presence of Cognitive impairment	0.63
Neuropsychological testing: Reduced MMSE	0.34
Neuropsychological testing: Improvement in MMSE Post LP	0.32
Neuropsychological testing: No Improvement in MMSE Post LP	0.50
Neuropsychological testing: Abnormal Demtect	0.11
Neuropsychological testing: No Improvement in Demtect Post LP	0.36
Neuropsychological testing: Intact Cranial nerves	0.54
Neuropsychological testing: Abnormal Cranial nerves	0.10
Neuropsychological testing: Presence of Psychomotor slowing	0.35
**Shunt type**	
Shunt Manufacturer: Hakim Medos	0.15
Shunt Manufacturer ProGav Miethke	0.15
Use of Shunt assistant	0.06
**Radiology**	
CT/MRI: Presence of Expanded internal CSF spaces	0.24
CT/MRI: Presence of Expanded external CSF spaces	0.66
CT/MRI: Presence of Global brain atrophy	0.27

Table 3 delineates the Chi-Squared test results for categorical variables analyzed against the response categories of iNPH patients post-ventriculoperitoneal shunt surgery. The asterisk (*) denotes statistical significance (p < 0.05)

**Fig. 3:**
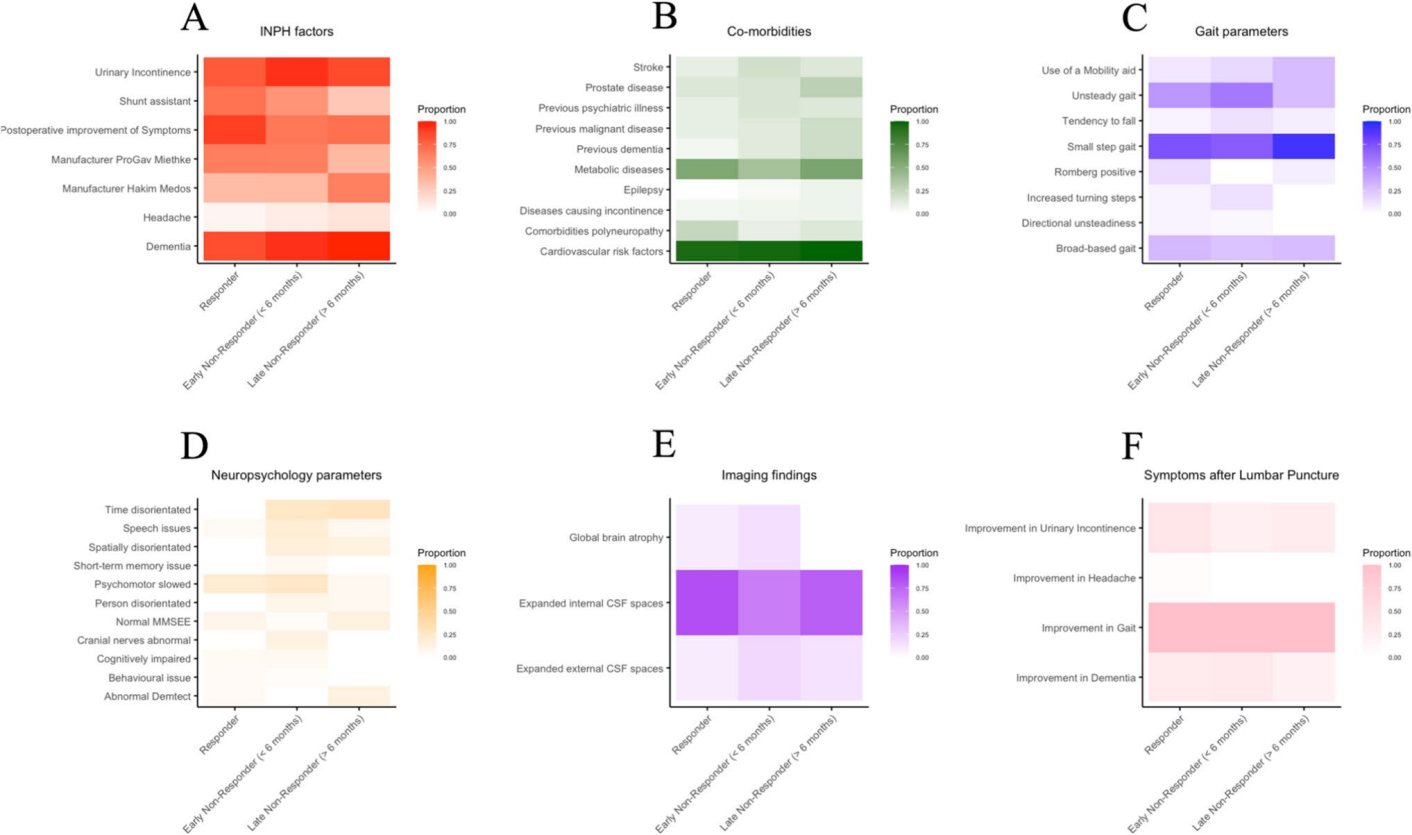
Heatmap analysis of categorical covariates by response category post-ventriculoperitoneal shunt surgery. Figure 3 depicts a panel consisting of six sub-figures 2 **A**-**F**, each respectively labelled alphabetically. Figure 3 provides a comparative visualization of various clinical factors associated with patient outcomes in idiopathic normal pressure hydrocephalus (iNPH) across different response categories (early non-responder (< 6 months), late non-responder (> 6 months), responder) to shunt surgery. The color gradient represents the proportion of patients within each category, with darker shades indicating a higher prevalence. Across all heatmaps, the x-axis represents the response categories ranging from early non-responders to late non-responders, including those who responded positively to treatment. Figure 3A examines postoperative improvement and other iNPH-related factors. Figure 3B showcases a range of comorbidities. Figure 3C focuses on various aspects of gait and mobility. Figure 3D highlights neuropsychological features. Figure 3E reflects imaging-related findings. Figure 3F highlights improvement of urinary incontinence, headache, gait and dementia after patients received a Lumbar Puncture between the three groups

The ANOVA analysis outlined in Table 2 showed there was no statistical significance in the interval between initial presentation and surgery, and the duration of symptoms between the response groups. Figures 3C and 3D explore the time intervals between presentation and surgery, and the duration of symptoms prior to surgery, respectively. Figure 3F indicates no significant differences in post-lumbar puncture symptom improvements across all groups, highlighting that this intervention does not discriminate based on response category, i.e. the type to degree of lumbar puncture response does not indicate overall improvement post surgery. Overall, there were no significant differences in co-morbidities or characteristics between the three groups other than time disorientation.

### Hakim triad

The distribution of gait impairments also showed variability among response categories, with broad-based gait observed in 26% of early non-responders, 30% of late non-responders, and 30% of responders. Figure 3C reveals that an unsteady gait is more common in early non-responders than late non-responders. Cognitive challenges, such as time disorientation, were documented in 29% of responders but were absent in late non-responders, this was a significant difference at *p* = 0.037.

Contrary to initial expectations, variables such as urinary incontinence and dementia, which are clinically significant in iNPH, did not demonstrate a statistically significant variance (*p* = 0.34434095 and *p* = 0.25435641 respectively), indicating a uniform prevalence across the three response groups. Figure 3D emphasizes neuropsychological factors, with speech issues being more pronounced in both early and late non-responders. Interestingly, the incidence of abnormal Demtect, a measure of cognitive function, is significantly higher in late non-responders.

### Radiological markers & shunt choice

Radiological findings indicated that internal CSF spaces were expanded in 66% of early non-responders, 70% of late non-responders, and 85% of responders, however there was no statistical significant difference, this is demonstrated in in Fig. 3E.

The choice of shunt device varied among the groups, with Hakim Medos devices being used in 37% of early non-responders, 70% of late non-responders, and 35% of responders, this did not affect outcome statistically. Figure 3A highlights the prevalence of shunt types and postoperative improvement. Notably, there's a higher usage of Hakim Medos shunts in late non-responders. Conversely, early non-responders and responders exhibit a more varied distribution of shunt types.

### Shunt valve changes

There was no significant difference in initial shunt pressure level between the three groups (Table 2), the mean initial shunt pressures (mmH20) were 93.4 in early non-responders, 107 in late non-responders and 99.5 in responders.

Overall, there were 131 pressure changes (31%), however only 17 (13%) adjusted were superseded by improvement. In early non-responders, there is a high number of valve adjustments, suggesting frequent postoperative interventions aimed at optimizing shunt function shortly after surgery. Late non-responders also show a significant number of valve changes, which may reflect later adjustments due to evolving symptoms or delayed identification of suboptimal shunt performance. Among responders, valve changes are observed in approximately half of the patients, indicating that shunt adjustments occurred even in those with a generally positive clinical outcome.

### Complications

There were 431 follow-ups appointments in total, over the entire follow-up period, there were 53 follow-up appointments where complications were identified, a complication rate of 12.2%. Thirty-nine of these were over-drainage, 2 were under-drainage; 25 hygromas were identified, 17 subdural haematomas, 8 underwent shunt revision.

## Discussion

Our result of 46.2% sustained improvement at 6 months fits within the established, albeit lower side, of published early shunt response literature in NPH [6, 19, 25]. The long term shunt non-response (early and late) fits well within established literature, Vanneste et al. found sustained response at 5 years only in 21% of patients, Savolainen et al. found 21–40% at 5 years, likewise Kahlon et al. 10–40% and Gencer et al. 35–80% at 3 years [9, 14, 25, 29]. The concept of long term failure in shunt response in NPH is not new, however causative factors of this phenomenon are not adequately explored. This paper aimed to delineate the transient shunt responder from the long-term shunt responder, and the distinguishing factors between them.

The categorization of shunt non-responders (S-NR) into early and late groups based on the onset of symptom deterioration post-surgery offers valuable insights into the temporal dynamics of the disease. Rather than viewing late and early non-responders as a uniform group, we observed that symptom trajectories in these patients follow varied patterns. Some patients demonstrate extended periods of stability postoperatively before experiencing sharp declines, while others exhibit consistent deterioration soon after surgery. This variability suggests that symptom progression is not a steady, linear process, but one that fluctuates, perhaps in response to underlying pathophysiological factors like cerebral ischemia or neurodegeneration [4].

Despite frequent valve adjustments in the two non-responsive groups, there was no discernible improvement, indicating it was not an issue with the procedure, rather with patient selection or surgical timing. Despite these repeated shunt valve adjustments, non-responders continue to deteriorate, with symptom deterioration at best slowed down in late non-responders, raising important questions about the underlying pathophysiological mechanisms that may render both valve changes and, indeed, shunt surgery itself ineffective. Although existing literature demonstrates higher rates of shunt adjustment (1.68 mean per patient) than our study, there is no established causative link between overall number of adjustments and patient outcome [18]. Despite adequate follow-up and and adjustment where necessary, there were still a significant proportion of transient responders who began to deteriorate. It is also important to note this cohort’s complication rate aligned with existing literature, complications rates tend to range between 20–40% further adding validity to results [20, 21].

Patient selection was in-line with recognised protocols, relying on objective CSF tap-test improvement, however recent analysis show that CSF tap test alone to be weak predictor of response, [27] interestingly our paper has shown that type and degree of tap test improvement did not discriminate between the groups, suggesting symptomology maybe not be the best predictor of long-term response.

One potential explanation relates to the pathogenesis of iNPH, which involves chronic cerebral ischemia caused by impaired CSF dynamics. The imbalance between CSF production and reabsorption leads to ventricular enlargement and mechanical compression of the periventricular white matter, particularly the corticospinal tract and other critical motor pathways. This process, especially in its earlier stages, is believed to cause reversible ischemic damage, which may improve with prompt shunt placement and proper pressure adjustments [15].

However, in non-responders—particularly early non-responders—it is plausible that more advanced disease has led to irreversible neuronal damage and increased brain stiffness, especially in the deep white matter and frontal subcortical areas. Magnetic resonance elastography (MRE) studies provide strong evidence for this, showing that cerebrum brain stiffness tends to increase with disease progression in iNPH [7]. This stiffness, likely associated with chronic ischemia and tissue compression, may lead to irreversible damage to neuroanatomical structures responsible for symptom improvement, such as those regulating gait, cognition, and urinary continence. MRE represents a useful diagnostic tool, as studies have shown stiffness to trend downwards in healthy controls with age [2]. As a result, these patients may never have benefitted from shunt surgery at all, as their brain tissue was already too damaged to respond to reduced pressure [17]. This theory aligns with previous clinical studies demonstrating that once these degenerative changes are established, shunt treatment—even when optimized with pressure adjustments—does not result in significant clinical improvement [12].

In early non-responders, it is conceivable that valve adjustments may still alleviate pressure in some ischemic regions, providing initial symptom relief. However, the persistence of neurological symptoms in these patients suggests that, despite adjustments, the underlying ischemic damage has progressed too far to be reversed [5]. This rapid decline is likely driven by an interaction between persistent ischemic changes and irreversible neuronal damage. Studies such as those by Klinge et al. support this view, revealing that improvements in cerebral blood flow after shunt placement are often limited in late-stage NPH, particularly in areas affected by chronic ischemia [16]. This may also explain the flaw in utilising CSF tap test as a means of selecting long term shunt responders.

A more compelling implication of our findings is that these early non-responders may represent a later stage of iNPH that does not benefit from shunt surgery. This group, particularly those identified in the first six months after surgery, could benefit from more sophisticated diagnostic tools like MRE prior to intervention. Early identification of these patients, using advanced neuroimaging to detect brain stiffness and ischemic damage, could save both patients and healthcare systems from unnecessary interventions and allow for a focus on exploring alternative treatment strategies—an area where further research is urgently needed.

A second theory, although less supported by our data, posits that non-responders, especially early ones, may suffer from concurrent neurodegenerative diseases such as Alzheimer’s or vascular dementia, which coexist with iNPH. This theory suggests that while shunt adjustments may alleviate the hydrocephalus component, coexisting degenerative pathologies persist, driving the non-responsiveness in this subset of patients [3]. This notion is further supported by the work of Freimann et al., who observed limited or no improvement in certain brain regions after shunt interventions in patients with concurrent neurodegenerative conditions [8]. However, our regression analysis showing no significant correlation between comorbid neurodegenerative conditions and symptom persistence makes this explanation less likely in our cohort.

Overall, the ineffectiveness of shunt valve adjustments in both early and late non-responders likely stems from the pathophysiological changes of iNPH itself. Chronic ischemic damage and increased brain stiffness in later stages of the disease may render the brain tissue incapable of responding to pressure reductions. These findings further underscore the importance of early intervention and precise diagnostics, such as MRE, to identify patients who may not benefit from shunt surgery and to focus on exploring novel treatment strategies to manage their symptoms. Understanding and predicting the late non-response phenotype in iNPH will likey require a multidisciplinary approach, integrating neuroimaging, neuropsychology, neurosurgery, and advanced computational models. Prospective longitudinal studies, involving in depth phenotyping and high-resolution neuroimaging, will be instrumental in exploring the temporal evolution of iNPH and the transition to late non-response. Such studies would not only shed light on the underlying mechanisms but also assist in the development of predictive models and targeted therapeutic interventions, tailored to the individual patient's pathophysiological profile.

### Limitations

This study's retrospective design and the reliance on clinical records introduce potential biases related to data completeness and the accuracy of symptom reporting. The single-centre setting may also limit the generalizability of the findings, and the categorization of non-responders based on time intervals, while useful for analysis, may oversimplify the complex and individualized nature of symptom progression in iNPH. Additionally, the regression analysis, while providing some insights, is constrained by the data's retrospective nature and the sample size, limiting the robustness and generalizability of the findings.

## Conclusion

In this cohort of 65 patients followed for a mean of 3.75 years after ventriculoperitoneal shunting, 54% were early non‑responders (< 6 months), 15% were late non‑responders (> 6 months), and ~ 31% were responders at last follow‑up. Routine comorbidity and radiological assessments did not differentiate responders from non‑responders. Valve adjustments (performed in 31% of cases) provided at best transient benefit in some late non‑responders and were ineffective in early non‑responders.

## Data Availability

The datasets generated and/or analysed during the present study include pseudonymised and anonymised clinical information. Due to ethical and legal restrictions, they cannot be made publicly available. Data may be shared on reasonable request to the corresponding author, subject to approval by the relevant institutional review board and in accordance with applicable data protection regulations.

## Code availability

Code is available upon request.

## Abbreviations

*AUC*: Area under the curve
*CA*: Callosal angle
*CSF*: Cerebrospinal fluid
*CT*: Computed Tomography
*CVS*: Cerebrovascular stroke
*DAG*: Directed Acyclic Graph
*EDA*: Exploratory Data Analysis
*FAMD*: Factor Analysis for Mixed Data
*ICP*: Intracranial Pressure
*iNPH*: Idiopathic Normal Pressure Hydrocephalus
*MMSE*: Mini-Mental State Examination
*MRI*: Magnetic Resonance Imaging
*QOL*: Quality of Life
*SD*: Standard Deviation
*S-NR*: Shunt Non-Responders
*VP*: Ventriculoperitoneal
